# Follow-Up of Advanced Parkinson's Disease Patients after Clinical or Surgical Emergencies: A Practical Approach

**DOI:** 10.1155/2020/8860785

**Published:** 2020-10-29

**Authors:** Hélio A. G. Teive, Matheus Gomes Ferreira, Carlos Henrique F. Camargo, Renato P. Munhoz

**Affiliations:** ^1^Movement Disorders Unit, Neurology Service, Internal Medicine Department, Hospital de Clínicas, Federal University of Paraná, Curitiba, Paraná, Brazil; ^2^Neurology Diseases Group, Postgraduate Program of Internal Medicine, Hospital de Clínicas, Federal University of Paraná, Curitiba, Paraná, Brazil; ^3^Movement Disorders Centre, Toronto Western Hospital, University of Toronto, Toronto, Ontario, Canada

## Abstract

**Background:**

Patients with advanced stage Parkinson's disease (PD) typically present with a myriad of motor and nonmotor symptoms in addition to comorbidities and, as a consequence, polypharmacy.

**Objective:**

To analyze a series of cases of advanced PD in which a clinical or surgical emergency played a trigger role in the irreversible progression of landmarks of the course of the disease.

**Methods:**

Data were collected during a 13-month observational period of a cohort of 230 PD patients, in 751 medical appointments. We included a total of 13 (5.65% of the total number) patients with advanced PD defined by Hoehn & Yahr (H&Y) stage ≥3 who presented with various clinical and surgical complications which, with the contribution of drug interventions, led to significant worsening of patients' overall clinical condition.

**Results:**

Hip fractures and infections were the most common complications identified. As part of this scenario, most patients presented with delirium, often requiring treatment with dopamine receptor blocking agents and/or had dopaminergic treatment withdrawn. Upon reassessment after 3 months, all patients remained bed or wheel chair bound (H&Y 5) and presented significant worsening of their UPDRS part III score of at least 10 points (mean 51.5 ± 3.3; paired *t*-test two-tailed *p* < 0.0001 compared to baseline). The mean dose of levodopa at baseline was 907.7 ± 149.8 mg (600–1200) and significantly higher (paired *t*-test two-tailed *p* < 0.0001) on follow-up, 1061.5 ± 175.8 mg (700–1300).

**Conclusion:**

Clinical and surgical emergencies are major determinants for a progression of PD to more advanced stages.

## 1. Introduction

By definition, patients with advanced stage Parkinson's disease (PD) present with a variety of symptoms derived from the underlying progression of the neurodegenerative process as well as from complications of therapy, most often in combination [1–3]. The rate of progression of PD, particularly in stages IV and V, is well known, from the classic study of Hoehn and Yahr, published in 1967 [4], to the seminal study by Kempster et al. [5] published in 2010. This scenario is further complicated by the coexistence of levodopa, refractory motor symptoms, and nonmotor signs to which treatment options are limited [1–3]. Moreover, due to advancing age, other variables, such as frailty, comorbidities, and polypharmacy, are almost always added to the equation [6, 7]. In addition, a series of emergencies that may occur in patients with PD can be considered, such as severe motor fluctuations and the “super-OFF” phenomenon, parkinsonism-hyperpyrexia and dyskinesia-hyperpyrexia syndromes, acute parkinsonism, and acute psychosis in PD, some of them caused by abrupt withdrawal of dopaminergic medications [8, 9]. The cumulative effect of this scenario is often translated into emergency scenarios such as infections, fractures, and vascular events. The clinical and occasional surgical management of these emergencies in patients with advanced PD requires sharp vigilance and knowledge of the underlying process to ensure that further insult is not to be added [2, 4, 6–9].

In this study, we present a series of cases of advanced PD in which a clinical or surgical emergency played a trigger role in the irreversible progression of landmarks of the course of the disease.

## 2. Methods

Data were collected during a 13-month (December 2018 to December 2019) observational period of a cohort of 230 PD patients, in 751 medical appointments, followed up in a large subspecialty clinic in South Brazil. The consecutive patients included met the following inclusion criteria: clinically confirmed diagnosis of PD as defined by standard criteria [10]; presenting with advanced disease defined by Hoehn & Yahr (H&Y) scale [4] stage 3 or higher in the OFF-medication condition [11, 12] (all selected patients had stage 4); and been clinically stable for the previous 6 months based on clinical judgment, which included been on regular treatment for the past two follow-up visits (every 3 to 4 months) with no overt psychosis or actively declining cognition having at least 10 years of clinical follow-up. Patients who were felt to be actively declining from baseline before any event were not included in the analysis. Formal patient inclusion occurred when presenting acute clinical or surgical emergencies leading to hospital admission. Informed consent forms were obtained by the patients by proxy, and the study was approved by the local ethics committee.

All patients were followed up and assessed by the same clinician (HT). The evaluation during hospitalization occurred upon the request of the physician conducting the case. All patients initially included were re-evaluated 3 and 6 months after the clinical and/or surgical emergencies. Specific data collecting protocol was used, including demographic information, type of aggravating clinical or surgical emergency event, longitudinal motor MDS-Unified Parkinson's disease Rating Scale (MDS-UPDRS part III) [13] performed in the off condition, and details of medication regimen, including mean daily levodopa dosage. Cognitive status was evaluated using the Mini-Mental State Examination (MMSE) [14] added by the Movement Disorders Society criteria for PD dementia and PD mild cognitive impairment (PD-MCI) [15, 16].

The results were presented as means, medians, minimum and maximum values, and standard deviations (quantitative variables) or as frequencies and percentages (categorical variables). The normality of the variables was evaluated by the Kolmogorov–Smirnov test. Student's *t*-test was used to compare groups in terms of quantitative variables. For the comparison of the groups in relation to categorical variables, the Fisher exact test was used. To evaluate the association between two quantitative variables, the Spearman correlation coefficient was estimated. Values of *p* < 0.05 indicated statistical significance. The data were analyzed using the IBM SPSS Statistics v.20.0 software.

## 3. Results

A total of 13 cases fulfilled the proposed inclusion criteria, corresponding to 5.65% of the total number of PD patients seen during the observation period of 13 months. The profiles of these cases are shown in Tables 1 and 2. Eight (61.5%) were male, the mean age for the whole group was of PD 76.8 ± 3.5 (72–85) years, and mean disease duration was 15.8 ± 2, 4 (13–20) years. Most cases of PD had the rigid akinetic subtype associated with tremor (80%) and only 20% without tremor. The mean off-period baseline UPDRS part III score was 39.8 ± 2.7. All patients fulfilled criteria for some form of cognitive dysfunction (Table 1).

The most common clinical event leading to hospital admission were an infection in 5 (38.5%) and femur fractured in 4 (30.6%) of cases. In 8 (61.5%) cases, a surgical procedure (abdominal or orthopedic) was eventually performed. The mean duration of hospital stay was 16.4 ± 5.7 (median 15), ranging from 10 to 30 days. None of the 13 cases died during the observational period. In addition to delirium, which was a feature of all patients, five cases also presented additional complications during their hospital stay, namely, cardiogenic shock, lung abscess, acute respiratory failure, sepsis, and acute peritonitis.

Upon reassessment after 3 months, all patients remained bed or wheel chair bound (H&Y 5) and presented significant worsening of their UPDRS part III score of at least 10 points (mean 51.5 ± 3.3; paired *t*-test two-tailed *p* < 0.0001 compared to baseline). The mean dose of levodopa at baseline was 907.7 ± 149.8 mg (600–1200) and significantly higher (paired *t*-test two-tailed *p* < 0.0001) on follow-up, 1061.5 ± 175.8 mg (700–1300) (Table 1). The increase in antiparkinsonian medication dose raised the individual levodopa equivalent daily dose (LEDD) between 2.1 and 34.1%. There was no correlation between and UPDRS before (*rs* = 0.106; *p*=0.72) or after (*rs* = 0.03. *p*=0.92) the acute events. There was also no correlation between the proportion of motor deterioration and need for dopaminergic medication increase (*rs* = −0.1308. *p*=0.67).

During admission due to their acute event, only two (15.4%) of the 13 patients were left with their dopaminergic treatment untouched, while the remaining had their therapeutic doses either reduced (4 cases, 30.1%) or stopped (7 cases, 53.8%). Among these 11 cases, once the change in dopaminergic treatment was detected by a consulting specialist, therapeutic approaches included reinstitution of standard oral levodopa therapy (4, 36.4%), use of a solution of dissolved levodopa via orogastric tube (4, 36.4%), or use of oral disintegrating levodopa sublingually (3, 27.2%). In addition, seven (53.8%) received doses of an antidopaminergic agent, particularly neuroleptics and antiemetics, and six (46.1%) required pain management with an opioid (Table 2). There was no statistical correlation between the use of antidopaminergic agents (*p*=0.07) or opioids (*p*=0.471) and worsening of UPDRS scores. The use of antidopaminergic agents resulted in an OD  =  0.27, less significant than reduction or withdrawal of levodopa (OD = 1.17) during admission, for a motor worsening of at least 25%.

## 4. Discussion

In this small observational study, we identified patients with advanced PD complicated by acute clinical or surgical events leading to important conclusions. Firstly, these serious intercurrences may potentially have a long-standing or permanent impact in patients' overall condition and well-being, representing a landmark in the progression of the disease in a quasistepwise fashion. This is probably not unexpected for this age range in general, but seems to be further aggravated by the underlying neurodegenerative condition, which, in fact, suffers a major hit as reflected by significant worsening of PD outcome markers (H&Y, UPDRS scores, and levodopa dose) even after complete resolution of the new clinical insult. Lastly, our other important observation was the fact that, in a good proportion of these cases, pharmacological management, including withdrawal and/or random changes in PD-related treatment and use of antidopaminergic agents, may have an added iatrogenic detrimental effect on clinical outcome.

A previous study found that morbidity and mortality in patients with advanced PD (H&Y > 3) is often related to a combination of disability due to the neurodegenerative process (levodopa refractory axial and bulbar symptoms (dysphagia, postural instability, and falls) and nonmotor symptoms (psychosis, dementia, etc.)) combined with a clinical complication, with sepsis or respiratory failure together occurring in more than 60% of these cases [17]. Pneumonia leading to sepsis is a widely recognized consequence of bulbar dysfunction and reduced mobility, while postural instability and falls lead to fractures and internal hemorrhages. PD patients have an almost four-fold increased risk of developing aspiration pneumonia when compared to the general population [18], while the prevalence of dysfunctional swallowing is higher than 50% [19]. Therefore, recognition of the potential additional harm added by a hospital admission due to falls, fractures, and infections, which can be a direct or indirect consequence of limitations derived from the advanced symptoms and signs of PD, raising the awareness of patients and caregivers, in addition to optimization of treatment is of fundamental importance.

Despite the observed impact of these clinical complications on mobility, as indicated by worsening of MDS-UPDRS III and H&Y scores, all cases presented with a hallmark of mental status deterioration with delirium as a major feature. This is clearly not a novel or unexpected feature as all, but one of these patients had some degree of cognitive impairment at baseline, most of them dementia. The correlation between these cognitive deficits and clinical complications is well known and illustrated in the study by Kempster et al. [5], a clinicopathological analysis of 129 PD cases of PD, which identified four milestones in the natural history of advanced PD, namely, frequent falls, visual hallucinations, dementia, and need for residential care. However, in a recent study, the authors confirmed that postural instability was the most frequently reported milestone across all monogenic parkinsonisms and may be most sensitive in marking the onset of advanced disease compared with cognitive impairment, dysphagia, and autonomic dysfunction [12]. The causes for admission in the context of PD have also been quite well explored in the literature. For example, a systematic literature review including a total of 7162 patients found that the main reasons for hospitalization were infections in 22%, primary worsening of PD symptoms in 19%, and falls and fractures in 18%, followed by cardiovascular comorbidities, neuropsychiatric, and gastrointestinal complications [20].

As highlighted previously in this manuscript, an important observation in our patients was the iatrogenic worsening of patient's condition due to either changes in dopaminergic treatment or use of antidopaminergic agents. This is a concern that was highlighted in previous studies. For instance, a study that tried to objectively quantify PD medication administration in hospitalized PD patients (93% of them in an internal medicine ward) found that almost 20% of medication doses were administered incorrectly, and among them, almost half were omitted. In addition, contraindicated medications were also frequently prescribed. Most of these mistakes occurred during the first 2 days after admission and when schedules were hospital-based as opposed to patient-based [21]. When patients were admitted to a surgical service, these figures are even more alarming: during an 18-month period, 71% had their PD medication doses omitted, more than one-third of them missing over 10% of prescribed doses. The values were similar for levodopa and dopamine agonists. In addition, antidopaminergic drugs (mainly antiemetics) were prescribed in 41% of cases. Complications, most commonly neuropsychiatric, were documented in 69% of these admissions [22]. Similarly, a study that assessed patients admitted to both clinical and surgical services showed that 74% had their PD medications stopped, omitted, or prescribed inappropriately. Among this group, 61% developed clinically significant complications attributed to the error, including transfer to the intensive care unit after abrupt medication withdrawal, falls, and profound akinesia also due to levodopa withdrawal. Another concerning finding was the fact that, in patients who became unwell as a result of their medications being withheld, there was a poor recognition and understanding of the whole situation by both doctors and nursing staff, for instance, profound akinesia mistaken for a stroke and failure to identify a dopaminergic drug. The study also identified poor knowledge about drugs that are contraindicated in PD patients with 11% receiving prescriptions for antidopaminergic medications. Finally, the study also found multiple instances in which rehabilitation therapy had to be cancelled due to severe akinesia as a result of patients not being given their medications [23]. As an answer to these issues, a prospective cohort study assessed the effect of implementation of a hospital-wide alert system incorporated into the electronic medical record system. The alert was triggered on three basic situations: admission of a PD patient, admission of any patient on dopaminergic therapy, and when these two types of patients were prescribed antidopaminergic medications. As the main effects of this intervention, the proportion of patients for whom contraindicated medications were ordered decreased by more than 40%, the frequency of medication administration with doses given over 30 minutes later decreased, and medications ordered correctly increased [24]. Additionally, with regard to often erratic and inattentive use of levodopa during these admissions, another complex situation is the fact that some of these patients required enteral feeding via oro-/nasogastric tubes, a route of administration not ideal for the standard use of levodopa and other PD medications due to problems with infusion and interactions with the diet content of proteins, limiting absorption and the effectiveness of this drug [25, 26].

Finally, as our study shows, other pharmacological agents may play a role in creating these scenarios, especially the frequent use of high potency analgesic drugs such opioids (more than one-third of cases in our series), which are known to increase the risk of an acute confusional state and delirium [27, 28]. The use of antidopaminergic drugs, such as risperidone, promethazine, haloperidol, chlorpromazine, and bromopride (more than half of cases in our series), as therapeutic interventions in patients suffering from a disease in which motor symptoms are widely recognized to be for the most part secondary to hypodopaminergic state, is concerning and warrants the need for more basic dissemination of essential details on the physiopathology of PD for the general practitioner [29].

Our study has limitations, mainly related to the small sample size, making a boarder generalization of our findings questionable. Additionally, we did not take into account variables such as the potential interference of other drugs (other than the ones discussed above) such as antibiotics, for instance. On the other hand, the strengths of our study included the prospective nature, assessment by the same movement disorders specialist at baseline and on long-term follow-up, and the systematic gathering of data over the study period.

In conclusion, patients with advanced PD are well known to be at risk for complications directly related not only to their motor (i.e., postural instability and falls) and nonmotor (i.e., acute psychosis) features but also to another collection of problems that are apparently not directly related to the disease per se (i.e., vascular and infectious complications). Although these are well-recognized parts of this equation [6, 7], in our case series, it highlights other important aspects: in patients with advanced PD admitted due to surgical or clinical complications, even if the treatment of the condition is seemingly successful, there is a significant risk of a stepwise drop in functionality and motor function as reflected by worse H&Y scale and UPDRS part III scores. Another aggravating angle of this scenario is the frequent introduction of iatrogenic components such as the neglect for the proper basic medication regimen used to treat the underlying neurodegenerative disease and the active use of drugs that interfere negatively with the patients' mental status (opioids and other analgesics) or with PD physiopathology (antidopaminergic agents). The present report can be used as a reminder for the need to proactively focus care and guidance on preventable complications, agile identification and management of the most common intercurrences, and vigilance to avoid elementary iatrogenic aggravations in patients already struggling with an intricate multifaceted disease and its complications.

For practical purposes, we have listed a useful guideline for physicians involved in the direct care of PD patients admitted due to clinical and surgical complications (Table 3).

## Figures and Tables

**Table 1:** Characteristics of advanced PD patients with clinical or surgical complications.

Variable	Before hospitalization	After hospitalization	*p* value^*∗*^
Gender—male (%)	8 (61.5)	—	—
Age, years ± SD	76.8 ± 3.5	—	—
Disease duration, years ± SD	15.8 ± 2.4	—	—
Hospital stay	16.4 ± 5.7	—	—
Mean daily levodopa dose (mg)	907.7 ± 149.8	1061.5 ± 175.8	**<0.0001**
Mean daily LED (mg)	1202.1 ± 197.1	1371.2 ± 219.4	**<0.0001**
UPDRS	39.8 ± 2.7	51.5 ± 3.3	**<0.0001**
Cognitive dysfunction, *n* (%)	13 (100)	13 (100)	
PD-MCI, *n* (%)	5 (38.5)	5 (38.5)	1
PD dementia, *n* (%)	8 (61.5)	8 (61.5)	
Mean daily rivastigmine patch dose (mg)	7.9 ± 2.9	7.9 ± 2.9	1

PD: Parkinson's disease; SD: standard deviation; LED: levodopa equivalent dose; UPDRS: Unified Parkinson's Disease Rating Scale: MCI: mild cognitive impairment.

^∗^Student's *t* test. Values of *p* <0.05 indicate statistical significance.

**Table 2:** Demographic and clinical description of advanced PD cases with clinical and surgical emergencies treatment.

Case	Age (years)	Gender	PD duration (years)	Acute event	Complication	Baseline off UPDRS III score	Levodopa dose	Other inhospital drugs	Levodopa management	Follow-up off UPDRS III	Follow-up H&Y scale
1	78	F	15	Acute diverticulitis	Delirium, acute peritonitis	43	Withdrawn	Bromopride	Sublingual	53	V
2	75	M	20	Femur fracture	Delirium	39	Withdrawn	Opioid, risperidone	Oral restarted	50	V
3	79	M	18	Broncho pneumonia	Delirium, lung abscess	43	Reduced	Opiod	Oral optimized	54	V
4	80	M	20	Acute myocardial infarction	Delirium, cardiogenic shock	44	Unchanged	Trimetazidine	None	55	V
5	77	F	16	Mesenteric thrombosis	Delirium	39	Withdrawn	Opioid	OG tube	59	V
6	74	M	14	Femur fracture	Delirium	38	Withdrawn	Opioid, levomepromazine	OG tube	48	V
7	73	M	17	Femur fracture	Delirium	42	Withdrawn	Opioid, promethazine	OG tube	52	V
8	72	F	13	Pulmonary embolism	Delirium, acute respiratory insufficiency	37	Unchanged	None	None	48	V
9	75	M	14	Acute diverticulitis	Delirium	38	Unchanged	Risperidone	Sublingual	50	V
10	79	F	16	Urinary tract infection	Delirium, sepsis	36	Reduced	None	Oral optimized	47	V
11	78	F	14	Femur fracture	Delirium	43	Reduced	Opiod, amlodipine	OG tube	53	V
12	74	M	13	Colonic pseudoobstruction	Delirium	38	Withdrawn	Neostigmine	Sublingual	52	V
13	85	M	15	Upper airway infection	Delirium	38	Reduced	Bromopride, haloperidol	Oral optimized	49	V

PD = Parkinson's disease; H&Y  =  Hoehn & Yahr stage, and UPDRS = Unified Parkinson's Disease Rating Scale.

**Table 3:** Basic recommendations for health care of physicians involved in the management of patients with advanced PD during evaluation of clinical and surgical emergencies.

1: never stop the use of levodopa abruptly
2: change on levodopa regimen (dose and intervals of administration) should only be done in specific situations following recommendations of a specialist
3: avoid the infusion of levodopa along the administration of enteral diet
4: avoid the use of typical neuroleptics
5: when use of neuroleptics is unavoidable, clozapine and quetiapine are the safer options
6: avoid the use of drugs with potential antidopaminergic effect (neuroleptics and antiemetics)
7: if GI prokinetic drugs are necessary, give preference to domperidone
8: be vigilant for drug interactions in elderly patients with PD and different comorbidities
9: as a last resort, dispersible levodopa may be used, sublingually
10: subcutaneous apomorphine can be used as a rescue medication, when available
11: if medication regimen needs to be adjusted, preference should be given to less essential drugs, such as MAO B inhibitors, dopamine agonists, and amantadine, preferably under the guidance of a specialist
12: anticholinergics should be strongly avoided to be used in this group of patients

## Data Availability

No data were used to support this study.
